# Juvenile Homicide Offenders Look Back 35 Years Later: Reasons They Were Involved in Murder

**DOI:** 10.3390/ijerph17113932

**Published:** 2020-06-02

**Authors:** Kathleen M. Heide

**Affiliations:** Department of Criminology, University of South Florida, 4202 E. Fowler Ave., SOC107, Tampa, FL 33620, USA; kheide@usf.edu; Tel.: +1-813-974-9543

**Keywords:** juvenile homicide offenders, juvenile murderers, follow-up study, recidivism, reasons for killing, motives, crime circumstances

## Abstract

Murders committed by juveniles have been a serious concern in the United States for more than 50 years. Decisions by the United States Supreme Court during the 21st century have reduced the likelihood that juvenile homicide offenders will be sentenced to life without parole (LWOP). As a result of these decisions, hundreds of prisoners who were sentenced as juveniles for murder to LWOP under mandatory sentencing statutes or its equivalent are now eligible for the reconsideration of their sentences. In light of these changes in sentencing policies and practices, follow-up research on juveniles convicted of murder is essential. This research is part of a 35-year follow-up study of 59 boys who were convicted of murder and sentenced to adult prisons in a southeastern state, and initially interviewed in the early 1980s. Twenty of these men agreed to participate in clinical interviews during which they reflected upon the reasons (i.e., motives, circumstances) for which they got involved in criminal behavior as juveniles. These reasons, which broadly tap tenets of psychological and sociological theories, were analyzed in terms of predominance. Thereafter, the attention focuses on looking at the relationship of these 20 reasons to recidivism among the 18 juvenile homicide offenders (JHOs) who have been released from prison. JHOs who lived in neighborhoods where crime was routine and who engaged in crime because the opportunity presented itself were approximately 20 and 22.50 times more likely to be arrested post release and returned to prison, respectfully. The implications of these findings, the limitations of the study, and suggestions for future research are discussed.

## 1. Introduction

Juvenile homicide has been “a red button issue” for decades in the United States [US] [1]. When youths are involved in murder, the public frequently asks why did these kids engage in this behavior and what should be done with them. These questions are particularly acute when the killers are young teens, come from “good families”, and/or the crimes are particularly horrific involving vulnerable victims, multiple victims, and/or multiple offenders.

In the last 50 years, the United States has seen two periods when murders by juveniles have shown escalating trends. The first one occurred during the period 1960–1975, when arrests for juveniles for murder and nonnegligent homicide increased by 200%, by far outdistancing the increase in the juvenile population during this time [2,3]. The second escalating trend in juvenile arrests for murders occurred between 1984 and 1993. During this period, the number of arrests of juveniles rose from 1004 to 3284. Moreover, the percentages of total homicide arrests involving juveniles more than doubled from 7.3% to 16.2% [4]. Notably, this significant increase in juvenile involvement in murder occurred at a time when the juvenile population was decreasing. Experts warned the nation to expect a wave of young “superpredators” [5] and forecasted that there would be a continued escalation in murders by juveniles in the forthcoming years when the juvenile population would be increasing [6,7].

In response to this dramatic upswing, legislators across the U.S. passed legislation in the late 1980s and 1990s making it easier to transfer juveniles involved in serious crimes, including murder, to adult court. Juveniles who were convicted in the adult criminal justice system during that period, like their adult counterparts, were subjected to capital punishment, life without parole (LWOP), and long prison sentences [4]. Over the next 30 years, many legal challenges to treating juveniles as adults, particularly when it pertained to the death sentence, were made in response to these policies with varying responses across the United States. 

In the 21st century, the United States Supreme Court has heard five cases involving juveniles convicted in the adult system and has provided significant relief to juveniles sentenced to death and life without parole. The Court recognized in these cases that science had established that juveniles are developmentally different from adults. Because their brains are not fully developed, youths under 18 are less equipped to critically evaluate situations and tend to be more impulsive than adults. They are also more subject to peer pressure and limited in their ability to extricate themselves from unfavorable home or neighborhood environments.

In 2005, the Court held in *Roper v. Simmons* that juveniles convicted of murder could not be sentenced to death under the Eighth Amendment’s ban against cruel and unusual punishment [8]. In 2010, in *Graham v. Florida*, the Court applied the Eighth Amendment protection to juveniles sentenced to LWOP for nonhomicides, such as kidnapping and robbery, forbidding that sentence under these conditions [9]. In 2012, the Court ruled in *Miller v. Alabama* that sentencing juveniles convicted of murder to LWOP under mandatory sentencing statutes constituted cruel and unusual punishment and held that juveniles sentenced to LWOP under these circumstances were entitled to sentencing reviews in which factors in mitigation must be considered [10]. In 2016, the Court held in *Montgomery v. Louisiana* [11] that the Court’s ruling in *Miller v. Alabama* applied retroactively. The Court’s decision in *Montgomery* meant that more than 2000 prisoners sentenced to mandatory LWOP as juveniles were eligible for resentencing [11].

It is important to note that the Supreme Court’s rulings in *Miller v. Alabama* did not preclude a trial court from sentencing a juvenile convicted of murder to LWOP. However, based on the Court’s recognition of children’s diminished culpability and their potential to change in this case, as well as in the prior *Roper* and *Graham* decisions, the Court believed that sentencing juveniles to LWOP would be uncommon. Justice Kagan explained, in writing the majority opinion, that it is extremely difficult to distinguish between a juvenile offender whose crime results from immaturity and the rare juvenile whose crime is indicative of an individual for whom growth and rehabilitation are not possible [10].

In the 2016 case of *Tatum v. Arizona*, the Supreme Court vacated the sentence of a juvenile homicide offender sentenced to LWOP even though the trial court judge noted the defendant’s age as a mitigating factor prior to imposing its sentence. The Court remanded the case back to the trial court, referring back to its language in *Montgomery v. Louisiana.* The Court reaffirmed that LWOP sentences should be imposed upon only “those rare children whose crimes reflect permanent incorrigibility” [12].

Interestingly, the Supreme Court’s rulings with respect to the sentencing of juveniles convicted of murder in adult court were decided as murders by juveniles in the U.S. were decreasing. The “bloodbath of violence” predicted for the 21st century by experts did not occur [13]. In fact, arrests of juveniles for murder has shown a decreasing trend since 1994 when juveniles comprised 16.7% of all homicide arrests [4]. Juveniles comprised 8%–10% of all homicide arrests from 2001 to 2010; from 2011 to 2018, they comprised less than 8% of all those arrested for murder [14,15].

Despite the large decrease in the proportionate involvement by juveniles in arrests for murder, juvenile homicide remains a controversial issue in light of the *Miller v. Alabama* decision. Legislators and courts across the U.S. have struggled to answer what sentence is appropriate for juvenile homicide offender (JHOs) who have previously been sentenced to LWOP for murder [16] and what legal procedures must be put in place for a fair hearing [17]. Appellate courts have had varying interpretations about whether the Miller decision applies to “de facto life without parole” sentences (e.g., 99-year sentence; three consecutive sentences of 30 years) or strictly to LWOP sentences [18,19]. In March 2020, the United States Supreme Court agreed to hear a case involving a juvenile murderer who was re-sentenced to LWOP by the trial court after his original LWOP sentence was vacated. In *Davis v. Mississippi*, the question before the highest court, which will likely be heard and decided during the 2020–2021 term, is whether the Eighth Amendment requires the trial court to make a finding that the juvenile is “permanently incorrigible”, before the sentencing authority may impose a sentence of life without parole [20].

Among the most pressing questions facing those who are tasked with the sentencing or re-sentencing of eligible JHOs, or their release in the case of parole boards, is how to decide whether the JHO can be safely released to the community at some future date or whether they are among the rarest of juvenile homicide offenders, “those whose crimes reflect permanent incorrigibility” [10], and are beyond rehabilitation. In recent years, scholars have investigated the recidivism of juvenile murderers looking for the correlates that distinguish JHOs who reoffend from those JHOs who do not. These studies provide valuable information about the characteristics of JHOs and their institutional experiences that are helpful in identifying risk.

To the author’s knowledge, there is no theory that specifically addresses juvenile homicide. Moreover, there is no study to date that has systematically explored with JHOs why they were involved in homicide. This study is a 35-year follow-up study of a subsample of male juvenile homicide offenders who were convicted of murder or attempted murder in the early 1980s. These “boys” were interviewed shortly after they were incarcerated in adult prison and again 35 years later by the author [21]. At the time of the follow-up interview, the subjects, who were men in their early 50s, were asked why they were involved in criminal activity, including murder, as juveniles. After responding generally, they were asked approximately 20 questions concerning their motives or the circumstances operating at the time of the homicidal incident. 

Although the formal testing of the specific theories of crime was beyond the scope of this study, the individual questions tapped broad tenets of psychological and sociological theories. These tenets were suggested in the initial interviews conducted by the author with the JHOs in the early 1980s. Thirty-five years later the participating adult JHOs were asked if broad constructs from seven sociological theories were factors in their criminal involvement: subcultural [22,23,24,25], social disorganization [26,27], strain [28,29,30,31,32], social control [33], labeling [34,35], radical criminology [36,37] and routine activities [38]. The men were also asked questions relating to psychological concepts in five areas including perceptions, thoughts or beliefs, emotional states, traits, and effects on behavior. These psychological constructs, as will be illustrated later in this article, are associated with cognitive theory [39], rational choice theory [40], moral development theory [41], trait theory [42,43], and behavioral theory [44].

This article examines the men’s responses in relation to their success or failure post-release. Asking individuals to reflect on their reasons for their involvement in murder as juveniles decades later may shed light on the factors that led them to participate in lethal behavior. In addition, their responses may provide insight into possible reasons that they reoffended. Accordingly, their answers may provide helpful information with respect to both prevention and intervention.

## 2. Literature Review

The literature on the recidivism of juvenile homicide offenders, although limited, has increased significantly in the 21st century with respect to clinical reports and empirical studies. Clinical accounts consisting of small sample sizes are particularly common when juveniles kill parents [45] or when juveniles are involved in sexual homicides [46,47,48].

Twelve studies were identified that reported recidivism rates of JHOs with sample sizes of 20 or more. With one exception, these studies were published in the 21st century; 10 of the 12 were published after the *Miller v. Alabama* decision. Follow-up periods for these studies ranged from one year to 35 years. Six of these studies examined JHOs confined in juvenile facilities [49,50,51,52,53,54]. The remaining six of these focused on JHOs committed to adult prisons [21,55,56,57,58,59].

### 2.1. Studies of JHOs Incarcerated in Juvenile Justice Facilities

Hagan [50] investigated the recidivism of 20 male juveniles convicted of murder or attempted murder, comparing it with 20 male juveniles convicted of nonhomicide offenses in Wisconsin. The follow-up period extended from five years to more than 16 years after release. Recidivism was nearly the same for the two groups; 60% of the JHO group and 65% of the control group were convicted of another crime post-release. Seven of the 12 JHOs who recidivated committed crimes against persons, but none were involved in another homicide [50].

Vries and Liem [54] followed up on 137 JHOs released from juvenile facilities in the Netherlands; their sample included both girls and boys. The follow-up period ranged from 1 to 16 years. Fifty-nine percent (n = 81) of the JHOs were convicted of a new offense during the follow-up period which ranged from 1 to 16 years. The recidivists were convicted of two completed murders, 16 attempted murders, and 123 other violent offenses. Significant predictors of recidivism identified after five years included age at first arrest, age at homicide, number of previous offenses, and relationships with delinquent friends [54].

Since the *Miller* case was decided, Trulson and his colleagues have conducted at least four published studies investigating the recidivism of JHOs and the correlates of reoffending. Trulson, Caudill, Haerle, and DeLisi [52] investigated the interrelationship between gang affiliation and commitment for a gang-related murder with respect to the recidivism of 1804 male serious and violent delinquents incarcerated in a southern correctional facility. This sample contained 338 youth incarcerated for a non-gang-related homicide and 126 youths confined for a gang-related murder. Overall, 50% of the entire sample was arrested for a felony over a three-year period. Multivariate analyses revealed that homicide offenders in general, gang-related homicide offenders, and gang members were significantly more likely to be rearrested for a felony when compared to other serious and violent delinquents. The odds of being arrested for a felony increased 28.5% for gang members, 72.10% for non-gang-related homicide offenders, and 89.4% for gang-related homicide offenders [52].

In a second study, 221 JHOs (offender sex not specified) who were committed to juvenile facilities under Texas’ blended sentencing structure and released after they had served the juvenile part of their sentences were followed-up by Caudill and Trulson [49]. During the 10-year post-release period, 58% of the JHOs were arrested for a felony. Three variables were correlated with higher recidivism: shorter time incarcerated, elevated observed program disruption scores, and assaultive behavior towards correctional staff [49].

In a third and larger study, Trulson and colleagues [53] examined the recidivism rates of 1400 male and female juveniles released from the Texas Youth Commission (TYC). Of the 238 juveniles who were committed to the TYC for homicide offenses, about 58% were rearrested within five years. The JHOs who were male, African American, and were involved in more ward assaults were at significantly greater risk of rearrest, compared to JHOs who were female, White, and participated in less assaultive behavior on the wards. JHOs who participated in the Capital Offender Program (COP), an intense treatment program, who served more time in the TYC, and did not have a record of assaulting other inmates had a significantly lower recidivism rate than JHOs who did not participate in the COP, had shorter stays, and engaged in assaultive behavior towards inmates [53].

In a fourth study, Trulson and Caudill [51] followed up three years later on 247 male and female JHOs released from the TYC. Thirty-five percent of the sample consisted of juveniles adjudicated for capital homicides; the remaining 65% were convicted of non-capital homicides. Fifty percent of the sample were rearrested within the three-year period; there were no differences in the recidivism of the two groups. Three factors were predictive of recidivism: JHOs who were neglected prior to state institutionalization and who were involved in assaultive behavior in confinement were significantly more likely to recidivate. In contrast, JHOs who served longer sentences were significantly less likely to reoffend [51].

### 2.2. Studies of JHOs Incarcerated in Adult Prisons

DiCataldo and colleagues [55] followed up on 22 JHOs (offender sex not specified) who served an average of 12.7 years in prison in Massachusetts. Notably, none of these offenders were transferred to adult court and received LWOP. Participants included two groups of JHOs: (1) those not waived to adult court and given a determinate adult sentence; and (2) juveniles automatically waived to adult court when the state law changed and convicted of second-degree murder or manslaughter. The average age of the sample subjects at the time of release was 29 years old. These men were at risk for an average of 7.8 years at the time of the follow-up study. Approximately 32% (n = 7) of these JHOs had a post-conviction offense; 18% (n = 4) of the sample subjects were convicted of a violent offense; none were convicted of another murder. Recidivists did not differ significantly from non-recidivists with respect to age at the time of release, total time committed, time at risk, community, family, weapons, and mental health problems. The researchers noted, compared to other studies, that recidivism in their study was low and speculated that the age at release might have accounted for the low recidivism rate [55].

Heide and colleagues [56] conducted five follow-up studies on 59 male JHOs convicted of murder, attempted murder, or manslaughter in adult court and sentenced to adult prisons in a southeastern state. In the first study, the post-release recidivism of the JHOs confined in adult prisons was similar to figures reported above with respect to their counterparts in juvenile facilities. During the 15–17-year follow-up, 73% (n = 43) of the 59 JHOs were released from prison despite receiving long sentences. The mean and median time these JHOs were at risk was 11 years, ranging from 1 to 16 years. Sixty percent of the 43 released JHOs were returned to prison, typically within three years for committing new crimes [33]. Three of those JHOs killed again [4].

Khachatryan, Heide and colleagues [58] subsequently conducted a second follow-up study of these 59 JHOs 30 years after their involvement in homicide. This study focused on release and recidivism using arrest data. During the 30-year period that ended in December 2012, the results indicated that three JHOs had died prior to being released. Of the 56 remaining, 48 (81%) were released despite having long sentences in many cases due to vastly different sentencing structures in the 1980s; only eight JHOs were still in prison at that time on the original homicide charges.

Of the 48 released JHOs, 42 (88%) were rearrested and 30 (63%) were rearrested for violent crimes during the follow-up period. The time at risk from release to first arrest for the 42 recidivists averaged 2 years and 6 months, ranging from 1 month to 17 years and 10 months. The time at risk for the six successes was longer than the time for the recidivists; it averaged 12 years and 5 months; it ranged from 3 years and 4 months to 26 years and 3 months. Two more offenders were known to have died after being released, bringing the number of deceased JHOs to five. Regression analysis indicated that JHOs who served shorter sentences (six years or less), relative to those who served longer sentences (seven years or more), were six times more likely to be rearrested for a violent offense. Demographic characteristics and pre-homicide criminal behavior were not related to post-release arrest [58].

In the third study, these same JHOs were classified into two groups based on whether the killing or attempted killing occurred during the commission of another crime (e.g., robbery, burglary) or during a conflict. Few differences distinguished the groups. Conflict-oriented JHOs were significantly more likely to use a firearm during the homicidal incident. Crime-oriented offenders were significantly more likely to have co-defendants. The two groups did not differ significantly with respect to other pre-incarceration variables, release from prison, number of post-release arrests, and number of post-release violent crime offenses [57].

In the fourth study involving the same sample subjects, several significant differences emerged between the JHOs who had accomplices (group JHOs) and the JHOs who operated by themselves (lone JHOs). Group JHOs, relative to lone JHOs, were more likely to be Black, to have a prior juvenile record and more prior juvenile arrests, and to be involved in a crime-related homicide and one that targeted a stranger. Compared to lone JHOs, group JHOs were more likely to be released from prison and to be rearrested. No significant differences were found, however, between the two groups with respect to the number of post-release offenses [59].

In the fifth study, Heide [21] followed up on these 59 JHOs 35 years after their commitment for murder or attempted murder with the intention of interviewing them. At the time of the follow-up, 10 of the original sample subjects were deceased and five could not be located despite repeated attempts. Of the 44 living subjects who were contacted by mail, 22 (50%) agreed to be interviewed about their experiences in prison and, if they had been released from prison, their experiences in the community.

Nineteen of these 22 JHOs had been released from prison during the 35-year period; 11 of them were returned to prison one or more times and were considered failures in this study. The mean time that these 11 men were in the community prior to arrest was 12 months; it ranged from 2 to 42 months. The remaining eight JHOs, considered to be successes, consisted of six offenders who had never been rearrested (average time at risk was 12 years and 3 months), two who had been rearrested each one time for minor violations and remained in the community (time at risk was 3 years and 9 months and 8 years and 5 months), and two JHOs who were released after 30 years and not rearrested (Miller cases, time at risk was 14 months and 60 months). Notably, the average length of time at risk for the successes was higher than for the failures [21].

Three variables significantly predicted success, which in this study was defined as not being returned to prison. JHOs who served longer sentences (15 years or more) and those who completed their high school equivalency diplomas (GEDs) were approximately 14 and 12 times more likely to be successful than JHOs who were incarcerated for shorter periods and did not complete their GEDs, respectively. In contrast, JHOs who went back to the old neighborhoods upon release were 93% more likely to be arrested and sent back to prison. Stated another way, JHOs who went back to the old neighborhood were 13.5 times more likely to fail than JHOs who did not return to their home neighborhoods [21].

### 2.3. JHO Recidivism: Summary and Conclusions

With the exception of the study by DiCataldo and colleagues, the recidivism rates of JHOs in the 12 studies reviewed were at least 50% and often noticeably higher. While the DiCataldo study may seem initially at odds with the others discussed, it is not. DiCalaldo attributed their sample’s overall lower arrests and arrests for violent crime to their JHOs being incarcerated for a relatively long time (12.7 years on the average) [32]. The average length of time JHOs served in DiCataldo’s study was indeed longer than the other studies discussed above. Notably, the length of time served was correlated or predictive of success in five of the studies reviewed above [21,49,53,55,58].

In the studies by Heide and her colleagues, the average time served by the 48 men released in the 30-year follow-up study was 8 years [57,58,59]. The average age of the JHOs at the time of the homicide in both Heide’s and DiCataldo’s studies was 16. In Heide’s studies, on average, the JHOs at the time of their release were less than 25 years of age, that is, approximately four years younger than the JHOs in the study by DiCataldo [55]. These findings suggest that longer sentences may be associated with greater maturity. It is also interesting to note that another variable, assaultive behavior in confinement, was correlated with post-release behavior in three studies by Trulson and colleagues [49,51,53].

The review of these 12 studies indicates that in investigating the correlates of recidivism, researchers have focused on demographic and prior record data, incident-related characteristics, institutional behavior, and post-release behavior. None of these studies asked JHOs about the reasons that they got involved in crime, including murder, approximately 35 years later. The current study is unique in that permits an investigation into the reasons that JHOs became involved in lethal behavior and the recidivism outcome. This study was designed to examine whether the reasons that JHOs participated in violent crime was correlated or predictive of post-release criminal behavior. If the motives and circumstances operating at the time these JHOs were involved in criminal behavior as youths were correlated with recidivism, it may be possible to devise intervention strategies to reduce the involvement of juveniles in violent crime and the post-release recidivism of adult JHOs.

## 3. Materials and Methods

### 3.1. Sample Subjects

The sample subjects were part of Heide’s original study and were in their early 50s when interviewed a second time [4,21,33]. The 35-year follow-up study was approved by the Institutional Review Board for the Protection of Human Subjects at the University of South Florida (Pro 00035814). As discussed in the literature review, 22 of the 44 original subjects who were alive and could be located consented to be interviewed [21].

### 3.2. Follow-Up Interviews

The semi-structured interview covered their experiences in prison and if applicable, their post-release and recidivism experiences. In addition, subjects were asked about their thoughts regarding why they got into trouble as juveniles. They were first asked what were the most important reasons for which they got into trouble. Afterwards, they were asked separately if a specific motive or circumstances was “not a factor”, “somewhat of a factor”, or “a big factor”.

This study used a mixed methods approach; it was designed to foster both qualitative and quantitative analyses by including both open-ended and closed-ended questions. For example, the open-ended question regarding why the adult JHOs thought they got into trouble as juveniles lent itself well to qualitative analyses, which will be reported in a subsequent article. Qualitative analyses allowed for the richness of data to be explored in the context of overall themes for sample subjects. The follow-up questions inquiring about specific reasons, which is the focus of this article, permitted quantitative analyses to be performed using an ordinal scale: 1 = not a factor, 2 = somewhat of a factor, and 3 = a big factor. Quantitative analyses made it possible to rank in order the specific motives or circumstances to determine their relative influence from the perspective of the sample subjects.

Although it was beyond the scope of this research to test individual theories, the motives or circumstances, hereafter referred to as reasons, were based on theoretical tenets from major criminological theories. The JHOs were asked if these reasons played a role in them getting into trouble as juveniles. The reasons could be grouped broadly into psychological and sociological themes, although clearly some variables spanned both categories.

The psychological concepts included perceptions, thoughts or beliefs, traits, emotional states, and effects on behavior. This study investigated 12 psychological concepts related to perception (being high on alcohol/drugs), thoughts or beliefs (crime just happened, crime as exciting and fun, an easy way to make money, crime paid, crime was not really wrong), emotional states (a conflict that got out of hand, feeling down, being angry, wanting revenge), traits (impulsivity), and the effects on behavior (no consequences for criminal behavior). These five constructs, as depicted in Table 1, are associated with five psychological theories, including cognitive theory [39], rational choice theory [40], moral development theory [41], trait theory (e.g., [42,43]), and behavioral theory [44].

The sociological concepts, as described in Table 2, included the societal influences that influenced a person to engage in criminal behavior. The nine sociological concepts explored in this study included tenets from subcultural theory (friends/peer pressure, gang involvement) [22,23,24,25], social disorganization theory (crime routine in the neighborhood) [26,27], strain theory (crime as a way to get material things one could not afford, needing money to buy drugs) [28,29,30,31,32], social control theory (little or nothing left to lose due to lack of societal bonds) [33], routine activities theory (opportunity to commit crime presented itself) [38], labeling theory (being labeled by society as a “bad kid”) [34,35], and radical criminology theory (a way to get back at society) [36,37]. Gang involvement was removed from the analysis after it was found that involvement in gangs by the JHOs was little to non-existent and had no relation to the homicidal involvement.

With the removal of the gang variable, 20 variables were left. A few JHOs did not know or did not understand the meaning of four variables: opportunity presented itself (n = 1), crime just happened (n = 1), a conflict that got out of hand (n = 3), and little or nothing left to lose (n = 1). Their responses to these items were coded as missing data.

The interviews averaged 4.85 hours. In one case, the author was not able to cover the reasons for crime involvement due to time restrictions occasioned by the subject needing to get back to work. In a second case, the subject denied involvement in the murder and any other criminal activity. Accordingly, the final sample size consisted of 20 subjects.

### 3.3. Data Analysis

Demographic and prior record information is provided below for the 20 JHOs who participated in the follow-up study and provided information on the motives and circumstances involved in their criminal behavior, including murder or attempted murder. Thereafter, case-related characteristics are briefly presented, including the prison sentences imposed and the time served on the original murder charge. Post-release arrest data were reported for the 18 JHOs who were released. These 18 JHOs were categorized as successes or failures based on them being rearrested and returned to prison.

Cross-tabular analyses were used to test for significant differences in the variables related to the JHOs’ reflections on the reasons for their criminal behavior as juveniles and post-release outcome. The 20 variables were dichotomized to indicate that (1) the tenet was not a big factor, or (2) the tenet was somewhat of a factor or a big factor. The significance level was set at *p* < 0.05. The Phi statistic was used to measure the strength of the association; the chi square risk estimate (odds ratio) was reported when the findings were significant. SPSS version 25 was used to conduct the analyses.

## 4. Results

### 4.1. Characteristics of JHOs and the Homicidal Incidents

Sixty percent of the 20 JHOs interviewed approximately 35 years after their involvement in the homicidal incident were Black; the remaining 40% were White. Fifty percent lived in high-crime neighborhoods as juveniles. The boys ranged in age at the time of the murder or attempted murder from 14 to 17 years with an average age of 15.8 (SD = 0.8). Eighty-five percent of the JHOs had prior arrests; 40% had arrests for violent crimes. The number of prior arrests ranged from 0 to 16, averaged 4.4 (SD = 4.9,) and totaled 89 for the 20 sample subjects.

In eighty percent of the cases, the JHOs acted with one or more accomplices and the victim died. Seventy-five percent of the homicidal incidents were crime-related; the remaining 25% were conflict-related. In 55% of these incidents, the victims were strangers.

Although 11 of the 20 JHOs were indicted for murder in the first-degree with many facing the death penalty, which was legal at the time, only four (20%) were convicted of that charge. More than half (55%) were convicted of second-degree murder, one was convicted of manslaughter (5%), and the remaining four (20%) were convicted of attempted murder.

Eighteen of the 20 sample participants were released during the 35-year period despite many having long sentences. The time served ranged from 25 to 421 months (35 years and 1 month) with an average of 151 months (12 years and 7 months) (SD = 119 months). Eleven (61%) of the JHOs served approximately from 2 to 9 years; the remaining seven (39%) served from 15 to 35 years.

### 4.2. Reasons for JHO’s Involvement in Criminal and Lethal Behavior

Table 3 presents the percentages of JHOs who rated the individual reasons as not a factor, somewhat of a factor, and a big factor in their criminal and lethal behavior using the three-point scale described above. The means and ranks of all 20 variables are also depicted in this table. The top four variables rated by the JHOs as constituting “a big factor” in the JHOs’ criminal involvement were friends/peer pressure, being high on alcohol and/or drugs, the crime “just happened,” and crime was routine in their neighborhood. The five variables that were rated the lowest in terms of being a “big factor” in the reasons for the JHOs’ criminal behavior were committing crime to get back at society, seeing their criminal behavior as not really wrong, lack of consequences from prior criminal behavior, trying to get back at someone, and needing money for alcohol and drugs.

### 4.3. Post-Release Failure of JHOs by Reasons for Criminal and Lethal Involvement

Cross-tabular analyses were conducted to investigate if failure on post-release was related to the 20 reasons for the 18 sample participants who were released from prison. Given the small number of cases, Fisher’s exact test values were reported, in addition to the chi square statistic, and were used to determine if the results were statistically significant. Two-sided tests were used because it was unknown how the reasons existing at the time that the JHOs were involved in homicidal incidents for would affect the post-release success, often many years later following their initial prison incarceration.

Two of the 20 variables were found to be significant. JHOs who reported that crime was routine in their neighborhood were significantly more likely to fail upon release into the community than JHOs for whom crime was not routine in their neighborhoods (chi square (1) = 6.199, Phi = 0.604, *p* = 0.013; Fisher’s exact test *p* = 0.028). JHOs who said that they were involved in crime because the opportunity presented itself at the time were significantly more likely to be arrested and sent back to prison than the JHOs for whom the opportunity to engage in crime was not a factor (chi square (1) = 6.804, Phi = 0.633, *p* = 0.009; Fisher’s exact test *p* = 0.035). The odds ratios for these two independent variables indicated that the JHOs who lived in neighborhoods where crime was routine and who engaged in crime because the opportunity presented itself were approximately 20 and 22.50 times more likely to be arrested post release and returned to prison, respectfully.

As noted in the literature review, Heide [21] found earlier that returning to the old neighborhood was predictive of the post-release failure for this same sample; JHOs who returned to the old neighborhoods were 13.50 times more likely to fail. Accordingly, additional analyses were conducted to see if living in neighborhoods where crime was routine as juveniles was related to JHOs returning to the old neighborhoods after release from prison. Cross-tabular analyses revealed that JHOs who lived in neighborhoods where crime was routine as youths were significantly more likely to return to the old neighborhoods when they were released from prison than JHOs who did not live in crime-ridden neighborhoods (chi square (1) = 6.199, Phi = 0.604, *p* = 0.013; Fisher’s exact test *p* = 0.028). The odds ratio indicated that the JHOs who lived in neighborhoods where crime was routine prior to their arrest for murder or attempted murder were 20 times more likely to return to these neighborhoods than the JHOs who did not grow up in this type of environment.

## 5. Discussion

To the author’s knowledge, this research is the first follow-up recidivism study of juvenile homicide offenders that included interviews with juvenile homicide offenders three decades after their involvement in the murder or attempted murder. At the time of the author’s second interview with the study participants, the JHOs were approximately 35 years older and were men in their early 50s with far more life experience. Moreover, this study is the only one to date that asked JHOs to reflect on the reasons that they engaged in criminal behavior that led to fatal or near fatal outcomes for their victims. Although the sample size was relatively small, several important findings emerged that warrant discussion particularly in the context of theoretical explanations for the JHOs’ criminal involvement.

One factor stood out as the most important reason that the JHOs got into trouble. Seventy (70%) percent of the men identified friends/peer pressure as a big factor in their criminal involvement with 20% more indicating that it was somewhat of a factor. More than 50% of the JHOs rated as big factors getting high, the crime just happened and crime was routine in their neighborhood; when the category is broadened to include somewhat of a factor, the percentages rise to between 69% and 80%.

Although this study was not designed to test specific theories of crime, these four variables include both sociological and psychological tenets that are consistent with subcultural theory (delinquent peers) [22,23,24,25], social disorganization theory (crime-ridden neighborhoods) [26,27], and developmental psychology (taking responsibility) [41,60,61]. Consistently with the subcultural and social disorganization theories, the findings paint a picture of teens “hanging out” and getting high in neighborhoods where crime is routine. Although using drugs and alcohol is typically a social activity for juveniles, these substances have known psychological as well as biological effects that are often amplified in teens: they impair judgment, affect perception, and may have a disinhibiting effect [62]. Clearly, crime does not “just happen”. However, when individuals are high, impaired by drugs and/or alcohol, associating with delinquent peers, and lacking in maturity, it may indeed seem that the crime just happened.

These explanations on the surface may suggest that the adult JHOs have externalized blame for their conduct by attributing their criminal involvement to social processes, peers, crime-ridden neighborhoods, and drugs. For some study participants, the failure to take responsibility for their behavior as juveniles is accurate and remains true 35 years after their homicidal behavior. For other adult JHOs, it is an indication of their insight into the reasons that they got into trouble, including murder, as youths. This issue will be systematically explored in a forthcoming manuscript that will analyze the data pertinent to the JHOs’ perceptions regarding their accountability for their homicidal involvement and their feelings about the victim(s) at two points in time: when interviewed as adolescents in prison and as adults in their early 50s. These data will provide information about three traits related to psychopathy, namely the failure to accept responsibility, the lack of remorse or guilt, and callousness/lack of empathy for the victim [43]. Examining data over time allows a preliminary assessment of the stability of these traits when present in JHOs.

Five factors were rarely identified by the men as big factors in their getting into trouble as juveniles: getting back at society, criminal behavior as not really wrong, not having consequences for prior criminal behavior, getting back at someone, and needing money for alcohol or drugs. The variables that were least likely to be endorsed as explanations tapped one concept in sociological theories, three in psychological theories, and one that spanned both. Getting back at society is consistent with radical criminology theory [36]. In contrast, believing that the crime was not really wrong, engaging in crime because of a lack of previous consequences for one’s behavior, and getting back at someone are consistent with psychological explanations, including moral development theory [41], cognitive theory [39], and behavioral theory [44]. Needing money for drugs can be an indicator of strain theory [31], behavioral theory (negative reinforcement to terminate an aversive physical and/or psychological condition, or positive reinforcement to bring on a positive state) [44]. To wit, few JHOs indicated that they participated in crime because they wanted to get back against society, because they did not see criminal behavior as really wrong, or because their criminal behavior had not been sanctioned in the past. Their remarks suggested that when “they did wrong,” they were not thinking in societal terms, knew their behavior was wrong, and had received some sanctions from the juvenile justice system in the past. Few men indicated that they engaged in criminal behavior because they wanted to get back at someone, that is, to seek revenge. Interestingly, only 25% said that they engaged in criminal behavior to get money for drugs or alcohol. Many said that they had access to drugs through friends or made money by working.

Only one third of the adult JHOs identified that they “acted impulsively” as a big factor in their criminal behavior. However, more than half (55%) said it was somewhat of a factor or a big factor in their criminal involvement. Impulsivity is another trait of psychopathy [43] and is a main factor in the self-control theory of crime, often referred to as the general theory of crime [63]. Interestingly, of the eight JHOs who were released and who identified acting impulsively as at least somewhat of a factor in their criminal behavior, four were successes and four were failures post-release. Clearly, no conclusions can be based on this small number of cases and more research on impulsivity and self-control with respect to JHOs is needed.

Given the small sample size, it is particularly meaningful to find that two variables, both from sociological explanations of crime, increased the likelihood of post-release failure. JHOs who lived in crime-ridden neighborhoods (social disorganization) [26,27] and who engaged in criminal behavior because they perceived that an opportunity was available to them (routine activities theory) [38] were approximately 20 and 22.50 times more likely to be arrested post release and returned to prison, respectively. These findings are particularly illuminated by a subsequent analysis that found that JHOs who lived in neighborhoods where crime was routine prior to their arrest for murder or attempted murder were 20 times more likely to return to these neighborhoods than JHOs who did not grow up in this type of environment; JHOs who returned to the old neighborhoods were 13.5 times more likely to be sent back to prison [21].

### Limitations of the Study and Directions for Future Research

The qualitative and quantitative nature of this 35-year follow-up study of juvenile homicide offenders’ reasons for involvement in criminal and lethal behavior was a noteworthy aspect of this research. The sample size of 20 is impressive for a study that spanned three decades and included half of the original sample subjects who were living and could be located. However, with that said, it was still small. The statistical power for the three chi square analyses that were significant was between 0.70 and 0.74, below the desired 0.80 power. Given the small sample size, the power achieved is unsurprising and was fairly close to the power standard preferred. Ideally, future studies should have larger samples, preferably of 100 subjects or more, to permit statistical analyses on multiple variables simultaneously.

This research was unique in that it asked JHOs as middle-aged men to reflect on their reasons for engaging in criminal and violent behavior as juveniles. It explored tenets of theoretical explanations of crime by asking the JHOs about their reasons for lawbreaking behavior. It would be valuable to do a more formal testing of the psychological and sociological theories given these preliminary findings. This study of JHOs was also unique in the length of its follow-up period. Clearly, a shorter follow-up period might be desirable, considering that 10 of the 59 sample subjects died within the 35-year period. A 20-year follow-up period would allow for maturational changes between those released before age 25 and those released after that time to be assessed in post-release success. A follow-up period of this length would enable the replication of findings that JHOs who served longer sentences are more likely to succeed than those who served shorter sentences [21,49,53,55,58].

Interestingly, a 20-year sentence is recommended by developmental psychologist, James Garbarino, who has evaluated dozens of JHOs who were initially sentenced to LWOP following the U.S. Supreme Court’s decision in *Miller v. Alabama* [10]. Based on his experience, Garbarino maintains that it takes this amount of time for the JHO to mature to the point that he is ready to recognize the need to change and to commit to, and engage in, a serious path to rehabilitation [64].

## 6. Conclusions

These findings, when taken as a group, clearly support the reasoning of the United States Supreme Court with respect to juvenile homicide offenders. As the Court noted, juveniles are much more likely than adults to be influenced by their peers and to be affected by the neighborhoods in which they life. Moreover, the results have direct implications for both prevention and intervention. They suggest that juveniles from crime-ridden neighborhoods need more adult supervision, safe places to go that provide alternatives to the streets such as a teen or recreational center, prosocial activities to engage in, and mentors. Parents, schools, churches, and other community organizations (e.g., Big Brothers and Big Sisters, the YMCA, Boys and Girls Club) need to help youths to take responsibility and to see themselves as accountable, and to provide evidence-based drug education programs.

JHOs in prison should be discouraged from returning to the old neighborhoods, particularly if their former homes were in communities where crime was common, and from associating with their old friends. They should be helped to find alternative placements in transitional housing in locales where they do not have friends involved in lawbreaking and using drugs. During their incarceration, JHOs should participate in re-entry programming that includes decision making and accountability. In addition, participation in evidence-based drug education programs should be mandatory if the individual used drugs prior to their confinement in prison.

Programs like the one suggested do exist. In Florida, the Community Transition Program (CTP) located at the Everglades Correctional Institution in Miami has a Lifers’ Program. Most of the men in this program have been convicted of murder; all have served at least 20 years in prison. Inmates are sent to this program, which has been run by Dr. Regina Shern for more than two decades, by the Florida Commission on Offender Review, formerly the Florida Parole Commission. Over its 24-year history, approximately 400 men have been released from CTP. The program has a 96% success rate. In closing, it needs to be remembered that some JHOs do succeed. The challenge is to provide the assistance to help others do the same. At the same time, research is critically needed to determine if it is indeed possible to identify, in the words of the United States Supreme Court, “those rare children whose crimes reflect permanent incorrigibility” [12].

## Figures and Tables

**Table 1:** Psychological explanations of crime.

PSYCHOLOGICAL CONSTRUCTS	WHY DO JUVENILES COMMIT CRIME?	VARIABLES
Perceptions	Cognitive theory: they commit crimes because their perceptions and/or emotions are affected by drugs or alcohol and contribute to them engaging in criminal behavior.	Being high on drugs or alcohol
Thoughts or beliefs are related to three psychological theories: rational choice, cognitive theory, and moral development theory. Some of the variables noted herein can apply to more than one of these theories.	Rational choice theory: they commit crimes because they believe that the expected benefits outweigh the potential costs.	An easy way to make money; Crime paid
	Cognitive theory: they commit crimes because they have irrational beliefs.	Crime just happened; Crime as exciting and fun
	Moral Development Theory: they commit crimes because they have low moral development.	Crime was not really wrong
Emotional states	Cognitive theory: they commit crimes because their thoughts or beliefs affect their emotional states that lead to criminal behavior.	A conflict that got out of hand; Feeling down; Being angry; Wanting revenge
Traits	Trait theory: they commit crimes because they have certain personality traits. Psychopathy is based on trait theory.	Acting impulsively
Effects on behavior	Behavioral Theory: they commit crimes because of the consequences that follow behavior. Positive reinforcement leads to a reward; negative reinforcement removes an aversive event; and punishment brings on a negative event.	No consequences for past delinquent behavior

**Table 2:** Sociological explanations of crime.

	WHY JUVENILES COMMIT CRIMES?	VARIABLES
SOCIOLOGICAL THEORIES		
Subcultural	They have differences in values and beliefs; they are influenced by the people with whom they associate.	Friends/peer pressure; Gang involvement
Social disorganization	They live in areas of the city where crime is concentrated due to weak institutions (churches, schools, social agencies), mobility, diversity of residents, female-based households, low income, welfare, and criminal role models.	Crime was routine in neighborhood
Strain	They do not have access to legitimate means to achieve success because of their socioeconomic status and do have opportunities to commit crimes.	Crime was a way to get material things one could not afford; Needing money to buy drugs
Social control	They do not have stakes in conformity; they lack attachment to conventional others (parents, teachers); they are not committed to or involved in prosocial activities (school, work, sports); and they do not endorse common values with respect to law-abiding behavior.	Little or nothing left to lose
Labeling	They have been labeled as bad kids or lawbreakers and act in accordance with the way they believe they are perceived, creating a self-fulfilling prophecy.	Being labeled by society as a bad kid
Conflict	They are poor and lack power so they commit crimes due to class conflict and economic and social inequities created by the rich and powerful.	A way to get back at society
Routine activities theory	They are motivated to commit crimes, and do so when they identify a suitable victim (vulnerable person) and a lack of guardianship (no one around, poor lighting conditions, lack of supervision).	Opportunity just presented itself

**Table 3:** Frequency, percent, mean and rank of the reasons for all 20 sample participants.

Reasons	Not a Factor	Somewhat of a Factor	Big Factor	Mean	Rank by Big Factor (n of Cases)	Not a Factor vs. Somewhat/Big Factor
Peers (n = 20)	2 (10%)	4 (20%)	14 (70%)	2.60	1	2 v 18
High (n = 20)	4 (20%)	5 (25%)	11 (55%)	2.35	2	4 v 16
Just happened (n = 19)	5 (26%)	3 (16%)	11 (58%)	2.32	2	5 v 14
Crime routine (n = 19)	6 (32%)	3 (16%)	10 (53%)	2.21	3	6 v 13
Exciting or fun (n = 19)	8 (42%)	2 (11%)	9 (47%)	2.05	4	8 v 11
Strain (n = 19)	7 (37%)	4 (21%)	8 (42%)	2.05	5	7 v 12
Labeled (n = 18)	8 (44%)	2 (11%)	8 (44%)	2.00	5	8 v 10
Conflict (n = 17)	7 (41%)	3 (18%)	7 (41%)	2.00	6	7 v 10
Opportunity (n = 19)	7 (37%)	5 (26%)	7 (37%)	2.00	6	7 v 12
Easy way (n = 16)	6 (38%)	4 (25%)	6 (38%)	2.00	7	6 v 10
Little to lose (n = 17)	8 (47%)	2 (12%)	7 (41%)	1.94	6	8 v 9
Impulsive (n = 18)	8 (44%)	4 (22%)	6 (33%)	1.89	7	8 v 10
Crime paid (n = 19)	9 (47%)	4 (21%)	6 (32%)	1.84	7	9 v 10
Feeling down (n = 18)	10 (56%)	1 (6%)	7 (39%)	1.83	6	10 v 8
Being angry (n = 20)	11 (55%)	2 (10%)	7 (35%)	1.80	6	11 v 9
Money drugs (n = 20)	13 (65%)	2 (10%)	5 (25%)	1.60	8	13 v 7
No consequences (n = 19)	12 (63%)	4 (21%)	3 (16%)	1.53	10	12 v 7
Revenge (n = 20)	14 (70%)	2 (10%)	4 (20%)	1.50	9	14 v 6
Not really wrong (n = 18)	12 (67%)	3 (17%)	3 (17%)	1.50	10	12 v 6
Get back at society (n = 18)	14 (78%)	1 (6%)	3 (17%)	1.39	10	14 v 4
